# Improving mental and neurological health research in Latin America: a qualitative study

**DOI:** 10.1186/1471-2458-9-334

**Published:** 2009-09-11

**Authors:** Fabián Fiestas, Carla Gallo, Giovanni Poletti, Inés Bustamante, Renato D Alarcón, Jair J Mari, Denise Razzouk, Sylvie Olifson, Guido Mazzotti

**Affiliations:** 1 Laboratorios de Investigación y Desarrollo, Facultad de Ciencias y Filosofía, Universidad Peruana Cayetano Heredia, Lima, Perú; 2 Departamento de Salud Pública, Facultad de Salud Pública y Administración, Universidad Peruana Cayetano Heredia, Lima, Perú; 3 Department of Psychiatry and Psychology, Mayo Clinic College of Medicine, Rochester, USA; 4 Department of Psychiatry, Universidade Federal de São Paulo, São Paulo, Brazil; 5 Honorary Visiting Professor, Centre for Public Mental Health, Health Services and Population Research Department, Institute of Psychiatry, King's College, University of London, London, UK; 6 Research and Programmes, Global Forum for Health Research, Geneva, Switzerland; 7 Departamento de Psiquiatría, Facultad de Medicina, Universidad Peruana Cayetano Heredia, Lima, Perú

## Abstract

**Background:**

Research evidence is essential to inform policies, interventions and programs, and yet research activities in mental and neurological (MN) health have been largely neglected, particularly in low- and middle-income countries. Many challenges have been identified in the production and utilization of research evidence in Latin American countries, and more work is needed to overcome this disadvantageous situation. This study aims to address the situation by identifying initiatives that could improve MN health research activities and implementation of their results in the Latin American region.

**Methods:**

Thirty-four MN health actors from 13 Latin American countries were interviewed as part of an initiative by the Global Forum for Health Research and the World Health Organization to explore the status of MN health research in low- and middle-income countries in Africa, Asia and Latin-America.

**Results:**

A variety of recommendations to increase MN health research activities and implementation of their results emerged in the interviews. These included increasing skilled human resources in MN health interventions and research, fostering greater participation of stakeholders in the generation of research topics and projects, and engendering the interest of national and international institutions in important MN health issues and research methodologies. In the view of most participants, government agencies should strive to have research results inform the decision-making process in which they are involved. Thus these agencies would play a key role in facilitating and funding research. Participants also pointed to the importance of academic recognition and financial rewards in attracting professionals to primary and translational research in MN health. In addition, they suggested that institutions should create intramural resources to provide researchers with technical support in designing, carrying out and disseminating research, including resources to improve scientific writing skills.

**Conclusion:**

Fulfillment of these recommendations would increase research production in MN health in Latin American countries. This, in turn, will raise the profile of these health problems, and consequently will underscore the need of continued high-quality and relevant research, thus fostering a virtuous cycle in the decision-making process to improve MN health care.

## Background

Although mental and neurological (MN) disorders account for about 13% of the global burden of disease, research in mental and neurological health has been chronically neglected, particularly in low- and middle-income countries. Not surprisingly, few efforts have been made in such countries -- including Latin American countries (LACs) [1,2] -- to devise strategies to overcome the challenges faced by this type of research. Initiatives to foster MN health research are urgently needed in order to systematically improve both health policies and clinical practice in LACs.

In a previous publication [2] we presented information resulting from interviews to key mental health actors in the Latin American region, focused on the status of MN health research. The basic purpose of that report was to generate a tool to sensitize health professionals, decision-makers and other stakeholders about handicaps in this area. The interviews also included questions about possible specific recommendations to overcome existing difficulties for MN health research in LACs. The interviewees' responses to this second set of questions are the theme of the present report. We employed qualitative methodologies to produce an initial but useful framework for evidence as it is still scarce for most low- and middle income countries, including LACs. [3-6]. This article presents concrete ways of dealing with the difficulties that MN health research faces in LACs, and intends to propose guidelines aimed at fostering effective MN health research.

## Methods

This study was part of an initiative by the Global Forum for Health Research and the World Health Organization (WHO) to explore the status of MN health research in low- and middle-income countries in Africa, Asia, and Latin America. A description of the methodology is presented below and in a series of publications [2,7].

Thirty-four professionals from 13 LACs were interviewed for this study. Fifteen were MN health researchers, 8 were stakeholders, and 11 were multiple-role actors (MRAs), or individuals who had worked in both the research and decision-making arenas. In establishing the sample frame, an effort was made to cover as many LACs as possible, and to balance the number of participants from each category (researchers, stakeholders and MRAs). Recruitment ended when the saturation criterion [6] had been reached -- that is, when no new data was being obtained. Researchers and MRAs were mainly identified through publications indexed in PubMed and PsycINFO, and stakeholders mainly through snowball sampling techniques. They were contacted by e-mail and, after agreeing to participate in the study, an interview was scheduled. All people contacted, except one, agreed to participate. Interviews were done either in person or by telephone. Table 1 describes the study sample frame in terms of country and category of participant.

**Table 1:** Description of sample by participant category and country

**Country**	**Researchers** **(n = 15)**	Stakeholders* (n = 8)	Multiple role actors** (n = 11)	**Total** **(n = 34)**
Argentina	1	--	--	1
Bolivia	2	1	2	5
Brazil	1	1	--	2
Chile	1	--	--	1
Colombia	--	--	1	1
Costa Rica	2	1	--	3
Dominican Republic	--	1	3	4
Ecuador	--	--	1	1
Honduras	1	--	--	1
Mexico	3	--	--	3
Panama	1	--	2	3
Peru	2	4	2	8
Venezuela	1	--	--	1

* Stakeholders included: (1) officials (decision-makers such as Ministry of Health officials, health insurers, legislators, donors, and research councils); (2) university administrators; (3) representatives from professional associations; (4) associations of users of mental health services; (5) other NGOs.

** Actors who had worked as both researchers and stakeholders.

The first author and a research staff member carried out the interviews. Both interviewers are MN health professionals with research experience and training in qualitative methodologies. To ensure consistency in the topics addressed across the interviews, an interview guide setting forth the areas and topics to be covered was developed [2]. These included the participants' background, their views about the status of MN health research activities, and their perception of factors facilitating and hindering both the production of research and the implementation of research results. In addition, the guide included questions about what recommendations participants would make for fostering MN health research production in their countries. The interviewers also probed for examples of where MN health was improved as a result of implementing research results. Interviews were recorded and then transcribed by a hired staff member. Each transcript was reviewed by the interviewer who conducted the interview.

The Institutional Ethics Committees of the Universidad Peruana Cayetano Heredia and the Universidade Federal de São Paulo reviewed and approved the study. Participants were apprised of the study's objectives and methodology and full confidentiality about their statements was guaranteed.

Transcripts were broken in extracts which were converted to a dataset. An extract is a block of sequential sentences found to contain a unique idea. As a first step, we identified extracts referring to our primary unit of analysis: recommendations to improve MN health research. Transcribed interviews were then read again to achieve a holistic impression of the data, and to begin to identify general themes that emerged in relation to the participants' background and circumstances (e.g., country context). This was followed by a careful reading of the identified extracts, which allowed us to propose a tentative coding scheme capturing key categories for classifying the recommendations offered in the interviews. Each extract was coded according to these key categories [8].

As coding progressed, new emerging themes were subsumed, when possible, within the already established key categories (i.e., the existing coding scheme). Nevertheless, new key categories had to be created on a few occasions. When this was the case, the extracts already coded were reviewed to assess goodness of fit with the revised coding scheme or list of key categories. In the end, recommendations to improve MN health research were further sorted into four major categories: i) capacity strengthening of human resources; ii) establishment of government-led strategies to facilitate a research culture; iii) establishment in local and international institutions - that is, at non-governmental levels -- of a culture for facilitating research; and iv) establishment of a sustained process of research production [2]. Table 2 lists the final classification of recommendations by type, or coding scheme employed in the data analysis, and shows the number of participants who spoke about each recommendation type as well as the related number of extracts.

**Table 2:** Summary of recommendations by recommendation type and category of participant

**Recommendation types**	**Researchers** **(n = 15)**	**Stakeholders** **(n = 8)**	**Multiple-role actors** **(n = 11)**	**Total** **(n = 34)**
**Capacity strengthening of human resources**				
Training	9 (12)	8 (15)	5 (11)	22 (38)
Interaction among actors	7 (10)	7 (11)	6 (10)	20 (31)
Incentives/rewards	6 (8)	1 (1)	3 (4)	10 (13)
				
**Establishment of government-led strategies to facilitate a research culture**				
Policies and standards	6 (7)	8 (12)	4 (8)	18 (27)
Empowerment of institutions	3 (4)	6 (10)	4 (5)	13 (19)
				
**Establishment in local and international institutions of a culture to facilitate research**				
Institutional culture	6 (9)	7 (12)	5 (9)	18 (30)
International support	3 (3)	3 (3)	2 (3)	8 (9)
				
**Establishment of a sustainable process of research production**				
Themes and methodologies	5 (8)	7 (14)	6 (11)	18 (31)
Funding	4 (5)	7 (11)	3 (4)	14 (20)
Diffusion of research results	3 (4)	2 (2)	3 (4)	8 (10)

Numbers in parentheses indicate the number of coded extracts pertaining to the identified recommendation type.

Two of the authors (FF and CG) independently identified and coded interview extracts. Inter-rater reliability ranged between 80% and 100% for each major and key category. There was consensus among all the authors in the final classification of the recommendations. In addition, data triangulation was performed to assess differences in the statements that could be dependent on the role of the participants, i.e. researchers, stakeholders or MRAs. In general, this analysis showed a good agreement across the three types of actors in the recommendations to boost research production and utilization of research products.

## Results

Participants discussed multiple ways of fostering MN health research and the implementation of its results. An overview of their specific recommendations for each of the 10 key categories is presented below, along with examples, when offered by the participants, of experiences where such recommendations were successfully implemented.

### 1. Capacity strengthening of human resources

Participants emphasized the need to improve the number and skills of human resources as an essential step to boost research and results implementation processes. Recommendations to achieve this goal were focused on training of health professionals, greater interaction among researchers, decision-makers and other stakeholders, and implementing a system of incentives and rewards for those involved in research.

#### Training

Participants recommended that training in three key areas should be part of health professional education, both at undergraduate and graduate levels: MN health issues, research methods, and methods for the implementation of research results. The participants agreed that MN health should be a required subject in the education of all health professionals, particularly physicians, irrespective of their specialization. This will produce professionals in all areas of health who would feel comfortable and interested in working on MN health issues. In this way, it would be possible to grow a critical mass of knowledgeable health professionals able to recognize needs in the MN health area, and translate them into relevant research ideas.

Regarding training in research methods, the participants agreed that this should be emphasized particularly for MN health professionals whose preparation in this arena has been neglected in comparison to professionals in other health areas. Lastly, the interviewees pointed out that training in results implementation methods, which has been largely absent in all health professional education programs (but especially so in the MN health field), would create a critical mass able to translate research findings into clinical interventions, programs and policies that can effectively benefit the population.

The importance of investing in human resources training is communicated by a Peruvian stakeholder who pointed out that the success of epidemiological studies, carried out in several Peruvian cities by the Peruvian Institute of Mental Health, was due, in part, to the development of psychiatry residents', psychologists' and other health professionals' knowledge in epidemiological methods: *" [The Institute] entrusted senior researchers to form and train research groups and work teams. This allowed the capacity strengthening of research groups, which eventually were in charge of supervising the work of new teams needed as the epidemiological studies were taking place in more cities."*

#### Interaction among actors

Participants argued that interaction between researchers, students and practitioners should be encouraged, and a special effort should be made to create opportunities for researchers to interact with decision-makers and other stakeholders as a way of enhancing the research production-results implementation cycle. By way of example, a Panamanian researcher reported on his success in getting research results translated into interventions thanks to his interaction with decision-makers who had the power to implement community interventions: *"I think part of the success that I have had in transferring the results of my study about depression in teachers into interventions is mostly due to the personal interaction I had with decision-makers. For this study, I organized a meeting to present the results to a group of invited teachers, school principals and the directors of the educational region."*

#### Rewards and incentives

Participants recommended establishing academic rewards and financial incentives for professionals engaging in research and translational work. Simple academic activities such as small within-institution meetings of professionals in all health disciplines, along with university students and faculty members, can serve as reinforcement mechanisms. Research activities and production should be strongly and effectively considered as criteria for academic ranking in residence programs and faculty promotions. In addition, a research-boosting package should include financial incentives for dedicating time to research work. Financial resources move human resources.

The impact of rewarding research production, even in small ways, is described by a Venezuelan researcher: *"In my institution, we have made some progress in research production, [...] part of it has to do with our initiative to reward research. For example, graduate theses in mental health are evaluated yearly, and the best ones are awarded with both a financial incentive and publication of the work in a local journal by the Venezuelan Society of Psychiatry."*

### 2. Establishment of government-led strategies to facilitate a research culture

Participants agreed that governments have a crucial role to play in stimulating research and translational activities in MN health. They offered a number of suggestions about how governments might do so, through the creation of pertinent policies and standards as well as through the empowerment of government-related institutions (such as National Institutes of Mental Health and public hospitals, among others).

#### Policies and standards

Government agencies need to raise the profile of MN health problems as public health priorities. This can be achieved through the generation and implementation of policies and legislation aimed to improve not only the health system, but also research activities in this area. An example of how government initiatives can favorably impact both health system and research activities was offered by an Ecuadorian researcher in talking about his government's efforts to improve the population's accessibility to MN health care services: *"An important first step to improve mental health in Ecuador has been the introduction of psychiatric services in all general hospitals; these services have broadened the range of opportunities for mental health professionals, including research activities."*

#### Empowerment of institutions

Governments can stimulate MN health activities and research through the empowerment of pertinent existing institutions (e.g., National Institutes of Mental Health). Strengthening of these institutions should start with actual and active recognition of the missions and objectives for which they were created, as well as consistent support of their activities. These steps should also include avoiding the duplication of effort and wasting of money and other resources that can occur when confusion exists about the specific roles and tasks that each institution should have and accomplish. Empowerment of institutions also protects MN health initiatives and research efforts from situations of political instability and helps to ensure their continuity.

The impact of one government's initiative to strengthen MN health institutions is described by a Peruvian stakeholder who spoke of the importance that the Peruvian government's political and financial support had in carrying out epidemiological studies in several cities in the country: *"The Ministry of Health made it possible that practically all research functions on mental health were transferred to the Institute [Peruvian National Institute of Mental Health]. For the last four years, this has allowed the Institute to carry out epidemiological studies in several cities. These studies, in turn, have allowed the identification of mental health priorities that have to be attended to in these locations."*

Other recommendations pertained to the creation of an office within health ministries to coordinate research initiatives and translation of their results. These offices would work closely with institutes, hospitals, international institutions, universities, donors and non-governmental organizations (NGOs), to organize activities, prevent duplication of effort, promote effective interactions and take consistent steps toward action. This coordinating role would also help to connect actors and institutions, create networks, disseminate information about MN health priorities, and motivate and facilitate support from the private sector, international agencies, universities and other academic centers.

### 3. Establishment in local and international institutions of a culture to facilitate research

Participants agreed that all types of health-related institutions should demonstrate a serious commitment to the consideration of MN health issues and nourishing an environment and administrative disposition that facilitates research and translational activities at intramural and extramural levels. Although most of the participants' recommendations pertain to all type of institutions (public and private), some are specific either to local- or internationally-oriented institutions.

#### Local institutions

Participants recommended that local institutions vigorously undertake to establish a "culture" favorable to both MN health issues and research activities. This culture encourages investment in activities that extend beyond the undergraduate and graduate training of health professionals to continuing education opportunities throughout their careers. Although universities would carry much of the responsibility in generating and promoting this culture, all types of health-related institutions, including, for example, professional associations, should be involved.

Health-related institutions should organize and present themselves as continuously engaged either in research-generating, research-promoting or research-disseminating activities. This can be achieved, in part, by using numbers of research publications as an indicator of efficiency and productivity, and by allowing their personnel to allocate time to these activities as part of their work responsibilities. Institutions can also promote multidisciplinary meetings, involving MN and non-MN health professionals such as psychologists, psychiatrists, neurologists, social workers, nurses, general practitioners, sociologists and epidemiologists. These meetings could take the form of workshops to identify needs for research evidence and generate ideas about how to find answers (e.g., identifying variables to study and methodologies to use). They would also serve as opportunities to involve new participants, as well as to communicate research results to the community. Ultimately, these activities will provide opportunities to de-stigmatize MN health issues and demystify the research process as a tool in addressing them.

Institutions should not allow limited resources to dissuade them from attempts to encourage intramural research activities. Even small manifestations of institutional research culture can have an important effect on the generation of research activities. An example is given by a Bolivian researcher: "*Many of the individual efforts to conduct research have been publicly promoted and academically recognized by the Bolivian Society of Psychiatry, since 1971. The fruits of this initiative have been seen particularly in the area of drug abuse and domestic violence."*

#### International institutions

While participants acknowledged that there are many international institutions already helping to support health research activities in LACs, they called for strengthening support given to research on MN health issues. They envisioned these institutions acting as catalysts for governments to channel resources toward research, and to facilitate the implementation and preservation of research policies. Moreover, international institutions can prompt their local counterparts and national governments to move into action, from the results of research to engaging in translational activities. Participants also suggested that international institutions, such as the World Health Organization, including the Pan-American Health Organization (PAHO), and high-prestige universities, among others, have a role to play in helping governments and local health institutes create interest in MN health research among health professionals. This support can be materialized by sponsoring courses on research methodology, lectures on advances in MN health research, the use of on-line resources or by providing assistance in pointing out priorities and research needs.

The appendix provides a case study example of how the participation and support of international institutions -- an American NGO working with a Brazilian university to evaluate a new alcohol consumption policy -- can help to make things happen.

### 4. Establishment of a sustainable process of research production

Participants agreed that strengthening all components of the research production cycle will increase research in LACs. Most recommendations focused on how to forge stronger links between components of this process, especially those seen to be the most vulnerable or the most likely to be effective in generating and translating research in LACs. These components include the generation of research ideas (themes and methodologies), writing proposals to obtain funding, writing reports or scientific articles and disseminating the knowledge gained from those studies.

#### Themes and methodologies

Participants observed that two factors are strongly related to the success of a research project, and to producing a set of results that inform policies, programs or interventions. The first factor is a focus on health problems that constitute local priorities. For example, for some LACs, domestic or interpersonal violence (as in the case study described in the Appendix) are topics able to draw widespread support, whereas for other LACs, structural violence or alcohol or illegal drug abuse problems are perceived as more important and thus are more able to attract support. The second factor is the use of epidemiological studies to generate population- or community-level information on the distribution and determinants of the problem, as well as local dimensions that need to be considered for its solution. A Mexican researcher commented on how having a focus on local priorities and using epidemiological studies ensures the interest of donors and supporting institutions: *"In Mexico, there is awareness of the importance of social and epidemiological investigations to deal with local priorities. This has led to closely collaborative work between the Ministry of Health, the Secretary of Health, and the WHO, leading to the achievement of the first National Survey of Mental Health"*

#### Funding

The participants also proposed strategies to increase funding for research projects. Several recommendations concerned encouraging researchers to search for funds from NGOs and the pharmaceutical industry. However, most of the interviewees, especially stakeholders, thought that governments should take the lead role in providing financial support to MN health research. This can be done through scientific and MN health institutions, encouraging them to direct funds to research in this area. Although research budgets in LACs are small, the study participants suggested that governments might, for example, devise mechanisms to diminish tax evasion, corruption and contraband, thus allowing money to be saved and devoted to health needs, including research. As a Panamanian stakeholder explained: *"In Panama, researchers have the possibility of applying for support from confiscated funds obtained from illegal drug trafficking, through the Comisión Nacional para el Estudio y la Prevención de los Delitos Relacionadas con Drogas (CONAPRE), which has made implementation of research projects in those areas possible."*

Governments can also play an important role in organizing and encouraging interaction between financial entities (national or international), universities, ministries and other governmental bodies, as well as in the training of human resources on how to identify funding sources, understand the requirements and terms of the funding opportunities and prepare research proposals.

#### Dissemination of research results

Dissemination of research results was identified as a pivotal factor, not only for the generation of further research but also for the translation and implementation of results into policies, programs or interventions. The first step in the successful dissemination of results is a well-written report or article. Participants agreed on the need to invest resources in developing researchers' scientific writing skills -- particularly in English. This can be achieved by activating partnerships and networks among local and international actors and institutions, as well as through the development of intramural writing programs and facilities in the institutions.

Participants suggested that locally generated research results might be disseminated in electronic and print versions by the local Academies of Science, NGOs, professional organizations, National Institutes of Health, and even health care institutions such as hospitals and clinics. These dissemination pathways can also be used to reach decision-makers. However, participants pointed out that the mass media may be a more effective way of communicating research results to them. As a general observation, researchers need to be more proactive in disseminating their findings beyond the research community and through the local media, effectively trying to "sell their products" to the decision-makers, as well as to the general public. As a Colombian researcher revealed: *"The results of my research are being used to design prevention programs in the city. This situation has been possible due to the immediate dissemination of the results. Every time we generate an investigation, it does not remain as a journal article only, we also call the local and national media to provide them the information to be divulged*." Such efforts speed and foster the process of translating research results into actual policies, programs or interventions. Given that dealing with the mass media requires a certain skill set, researchers could certainly benefit from training in this regard as well as in the writing oriented to a lay public.

## Discussion

This study collects ideas and recommendations to foster MN health research and the translation of its results in Latin American countries (LACs). These suggestions derive from in-depth interviews with 34 key actors from 13 LACs. To summarize, the recommendations comprise initiatives aimed at increasing human resources knowledgeable in both MN health issues and research matters, as well as engaging governments and pertinent local and international institutions in both endeavors. These initiatives include increasing the participation of stakeholders in the generation of research, and taking advantage of the interest of local (e.g., Ministry of Health) and international (e.g., WHO, governments of high-income countries) institutions in specific MN health issues (e.g., drug use, violence) and research methodologies (e.g., epidemiological studies).

Other recommendations offered by the study participants include implementing a system of academic recognition and reward to attract professionals to research and translational activities in MN health, as well as creating resources within institutions to provide researchers with technical help in designing, carrying out and disseminating research. This technical help should include intramural and other resources for the acquisition and improvement of scientific writing skills. These efforts would lead to well-designed and well-written research proposals, reports and scientific papers which can, in turn, facilitate further funding as well as the dissemination of research results through a variety of means, including the mass media.

While an important goal in itself, boosting research production can be one of the important steps that, along with the enhancement of other factors like political will and general public sensitization, can lead to an overall improvement of the MN health field and public health work, including accessibility to effective prevention and high-quality care. As shown by many reports, MN health is currently not a priority, especially in poor countries. This fact is most likely caused by a historical and misguided view that MN health has a small impact on the well-being of populations, which in turn leads to the view that MN health is not worth investing in [9-11]. This disadvantageous situation could be overcome if research data are generated to demonstrate to decision-makers, and also to the general public, that MN health problems are real and have a tangible and pervasive effect not only on individuals but on public well-being as well [12].

Solid research would not only help to position MN health as a priority, but also help actors to manage MN health problems efficaciously. Programs, policies and interventions would have a greater likelihood of effectiveness, efficiency and sustainability if they were evidence-based and therefore independent of the decision-makers' personal beliefs or pressures of the policy-making process. Evidence-based initiatives are likely to experience greater success since they permit researchers, stakeholders and decision-makers to deal with MN health problems in a locally appropriate manner. Epidemiological data, for instance, would provide government decision-makers and public policy planners with information about how many people are suffering from a specific health problem (quantifying the problem), who is suffering with that problem (locating the problem), and how it can be solved (identifying ways for control and prevention of the problem). Such information allows for mindful management of health issues [13]. Thus, the availability of research results permits health actors to confidently allocate resources and efforts to deal with MN health issues, as they have done with other more visible and better studied health problems such as tuberculosis [14]. Furthermore, research could help to set up a robust frame allowing MH health data serve as indicators that allow health actors to monitor the overall health status of the population.

Some of the recommendations that emerged in this study have been voiced elsewhere, although not specifically in the context of LACs [15-17]. This agreement with the still scant literature on the topic lends credibility to the findings presented in this report. This consistency with previous literature is important given the findings' susceptibility to bias due to the small sample size and the convenience-driven sampling technique used. The key contribution of this study thus lies in providing locally generated information on the need among health actors to select and engage in activities applicable to LACs for boosting MN health research and the translation of research results into policy and action.

It is also important to note many LACs are already poised to undertake many of the recommendations made by the participants. For example, universities and health institutions have a cadre of trained professionals who may be directed to get involved in MN health research. These individuals would eventually become leaders in training future generations of MN health researchers. Relatively inexpensive coordinating work by health institutions can stimulate useful and productive interactions among researchers, decision-makers, donors, and other supporting persons, entities or institutions, thus creating numerous opportunities for fruitful collaboration.

Existing MN health datasets from LACs represent another resource waiting to be used. It is possible, for example, to implement data sharing policies that would make datasets from government-funded projects publicly available. Such policies might also encourage non-governmental and private research institutions, in the spirit of philanthropy, to share their own datasets. As seen in several high-income countries, public data allow undergraduate and graduate students in the health professions and health professionals to boost the training and research production processes, as well as advance evidence-informed decision making processes.

Initiatives like those described above would need to be implemented in a manner that permits further evaluation [18]. Multilevel designs, for example, can be useful in quantitative evaluations of these kinds of initiatives [19,20]. This is because multilevel research methods are particularly suited to assessing simultaneously the effects of contextual variables (e.g., a new policy delivering research training grants, or encouraging public access to datasets) and individual-level characteristics (e.g., professional background of the beneficiaries of such policy), on given outcomes (e.g., the annual rate of publications by individual or institution). These methodologies have already been implemented to answer MN health questions such as those related to the link between mental disorders and social disadvantage [21], and we envision the use of these methodologies to evaluate initiatives to boost research production and results utilization.

## Conclusion

Because of the scarcity of resources in LACs, support for research efforts is crucial in efficiently improving the quality, efficiency and accessibility of MN health care. The participants in our study have outlined a number of recommendations for boosting MN health research production and translation. These are offered as starting points that willing and diligent health actors should consider when trying to enhance MN health research activities in Latin America. They include: increasing trained human resources; encouraging interaction among actors; promoting the interest of local, national and international institutions in MN health issues; making governments the main users of research results; valuing a culture of research; broadening the dissemination of research-generated knowledge; and stimulating a culture of evidence-informed policies. Together, initiatives such as these would raise the profile of MN health problems in Latin American countries and the awareness for more research in this area, improve the quality and relevance of the research undertaken, and lead to greater utilization of research evidence in decision-making to genuinely improve MN health care in the region.

## Abbreviations

LACs: Latin American countries; MN: mental and neurological; MRAs: multiple-role actors; NGOs: non-governmental organizations; PAHO: Pan-American Health Organization; WHO: World Health Organization

## Competing interests

The authors declare that they have no competing interests.

## Authors' contributions

GM, JJM, SO, CG and FF designed the study. GM, JJM, CG, DR, IB, GP and FF collected data for the study. FF and CG analyzed the data. FF wrote the first draft of the paper. CG, SO, IB, RDA, JJM, DR and GP contributed to the interpretation of the data and to writing the paper. All authors read and approved the final manuscript.

## Appendix

### Use of research to inform and evaluate a law to reduce alcohol-related interpersonal violence in Brazil

The role of research in implementing successful public health interventions, along with the important role that international institutions can play in working together with local institutions to support health research, is exemplified in this case study. It also illustrates the relevance of other recommendations offered by the participants as ways to boost MN health research. In particular, it underscores the importance of political willingness to make an accurate diagnosis of a public problem and to build an evidence-based plan for its solution -- a solution that includes the participation of many different actors and the use of creative initiatives to implement the interventions and assess their impact on the population.

The setting is a Brazilian city close to São Paulo, where violence was felt to be a significant problem but very little was known about its characteristics or its determinants. In this context, the Social Defense Secretary, at the request of the city mayor, asked the municipal civil guard to look at patterns in criminal activity/behavior in the city. This research showed that most violent acts were committed during night hours and were largely associated with alcohol consumption. With this knowledge, the city government decided to pass a law prohibiting the sale of alcoholic beverages after 23:00.

City leaders sought the full participation of the community and its leaders, as well as bar owners and their regular clients in promoting and complying with the new law. The campaign to disseminate information about the law included distribution of attractive and colorful brochures describing it and the reasons for its implementation, in simple language. Announcements were also made on the radio and placed in local newspapers. In addition, meetings were held with community leaders. A follow-up poll revealed that 98% of the city residents knew about the law and 93% supported it.

After the law was implemented, acts of interpersonal violence and police calls for service decreased, and citizen perception of personal safety and community order improved. In 2004, the local university and Pacific Institute (an American NGO) undertook, in cooperation with city officials, an independent evaluation of the new alcohol policy. Fully funded by the Fundação de Apoio à Pesquisa do Estado de São Paulo, the study showed a reduction in murders and assaults against women, attributable to the implementation of the policy.

This experience brings hope to other communities in Brazil seeking to prevent violent acts, and provides them with a model for doing so. It demonstrates that local communities have the power to prevent alcohol-related violence. Findings from the study were distributed to various Health Department authorities. Twenty-seven cities of the metropolitan region of the state of São Paulo subsequently adopted the same policy, and other important Brazilian cities are following suit. The researchers intend to write a book for the general public and government authorities about this experience and Brazilian public policy on alcohol control. This publication will be written in simple language and will describe the details of the experience, including policy implementation, enforcement and impact.

## Pre-publication history

The pre-publication history for this paper can be accessed here:


